# Antiinflammatory constituents of *Atractylodes chinensis* rhizome improve glomerular lesions in immunoglobulin A nephropathy model mice

**DOI:** 10.1007/s11418-019-01342-3

**Published:** 2019-07-03

**Authors:** Toshinari Ishii, Tetsuya Okuyama, Nao Noguchi, Yuto Nishidono, Tadayoshi Okumura, Masaki Kaibori, Ken Tanaka, Susumu Terabayashi, Yukinobu Ikeya, Mikio Nishizawa

**Affiliations:** 1 Department of Biomedical Sciences, College of Life Sciences, Ritsumeikan University, Kusatsu, Shiga Japan; 2 College of Pharmaceutical Sciences, Ritsumeikan University, Kusatsu, Shiga Japan; 3 Research Organization of Science and Technology, Ritsumeikan University, Kusatsu, Shiga Japan; 4 Department of Surgery, Kansai Medical University, Hirakata, Osaka Japan; 5 Laboratory of Pharmacognosy and Medicinal Resources, Yokohama University of Pharmacy, Totsuka-ku, Yokohama, Japan; 6 Center for Supporting Pharmaceutical Education, Daiichi University of Pharmacy, 22-1 Tamagawa-cho, Minami-ku, Fukuoka, 815-8511 Japan

**Keywords:** *Atractylodes chinensis* rhizome, Atractylodin, Nitric oxide, Kampo medicine, Immunoglobulin A nephropathy

## Abstract

The crude drug *Sojutsu*, as defined by the Japanese Pharmacopoeia, is the rhizome of *Atractylodes lancea* De Candolle, *Atractylodes chinensis* Koidzumi, or their interspecific hybrids (Asteraceae). *Sojutsu* is one of the traditional Kampo formulas, which are administered to patients suffering from stomach disorders, edema, and nephrotic syndrome. Although antiinflammatory effects of *Sojutsu* have been reported, its effects on the liver and kidney have not been extensively investigated. Here, we used a *Sojutsu* sample identified as *A. chinensis* rhizome and isolated several constituents from its ethyl acetate (EtOAc)-soluble fraction that decreased production of the proinflammatory mediator nitric oxide (NO) in interleukin 1β-treated rat hepatocytes. Among the constituents in this fraction, atractylodin showed the highest activity to suppress NO production, whereas hinesol, β-eudesmol, and α-bisabolol showed low activity. Atractylodin decreased the levels of inducible nitric oxide synthase, tumor necrosis factor α, and lipocalin 2 messenger RNAs (mRNAs). The EtOAc-soluble fraction of the *A. chinensis* rhizome extract was administered daily for 20 weeks to high immunoglobulin A (HIGA) mice, whose pathological findings resemble human immunoglobulin A nephropathy. This fraction decreased the weight of white adipose tissue and decreased mesangial proliferation and immunoglobulin A deposition in glomeruli. These results indicate that the EtOAc-soluble fraction, which included antiinflammatory constituents, may be responsible for improvement of the mesangial lesions in HIGA mice.

## Introduction

*Atractylodes* species (Asteraceae) are used as medicinal herbs in East Asia, and four *Atractylodes* species are used as crude drugs in Japanese Kampo medicine. These species are classified by the Japanese Pharmacopoeia into two crude drugs, *Sojutsu* and *Byakujutsu* [1]; *Sojutsu* (“Atractylodes Lancea Rhizome”) is defined as the rhizome of *Atractylodes lancea* De Candolle, *Atractylodes chinensis* Koidzumi, or their interspecific hybrids (Asteraceae), whereas *Byakujutsu* (“Atractylodes Rhizome”) is defined as the rhizome of *Atractylodes japonica* Koidzumi ex Kitamura or *Atractylodes macrocephala* Koidzumi (= *Atractylodes ovata* De Candolle). The *Atractylodes* species of *Sojutsu* samples can be discriminated by the nucleotide sequence of internal transcribed spacer 1 (ITS1) located between the *18S rRNA* and *5.8S rRNA* genes [2, 3].

*Sojutsu* contains sesquiterpenoids (e.g., β-eudesmol, hinesol, β-selinene, elemol, and α-bisabolol) and polyacetylene compounds (e.g., atractylodin and atractylodinol) [4–6]. The phytochemical content of these constituents differs in each *Atractylodes* species; For example, β-eudesmol and atractylodin are abundant in *A. chinensis* rhizome, whereas atractylodin is not detected in *A. macrocephala* rhizome [7].

The antiinflammatory effects of *Atractylodes* species rhizome extracts and their constituents have been reported. They inhibit production of proinflammatory mediators, including prostaglandin E_2_ and nitric oxide (NO), which are primarily produced in hepatocytes and macrophages and play a pivotal role in a variety of diseases [8, 9]. Indeed, *Sojutsu* has been used as a component of Kampo formulas, such as *Heiisan*, to treat stomach disorders including acute gastritis. Some extracts and constituents were investigated using RAW264.7 macrophages treated with the bacterial endotoxin lipopolysaccharide (LPS) [7, 10, 11]. However, there are few published reports on the pharmacological effects of *Atractylodes* species extracts or of each of their constituents on the liver or hepatocytes. Because the induction of inducible nitric oxide synthase (iNOS) mimics an inflammatory response [12], suppression of NO production is correlated with antiinflammatory activity. Therefore, NO has been used as a marker to estimate the antiinflammatory activity of Kampo formulas, i.e., *Saireito* [13], and their constituents, i.e., gomisin N in fruit of *Schisandra chinensis* [14], limonin in bark of *Phellodendron amurense* [15], and sakuranetin in bark of *Prunus jamasakura* [16].

*Goreisan* and *Saireito* are Kampo formulas containing *Sojutsu* and improve edema and nephrotic syndrome through diuretic properties. The high immunoglobulin A (HIGA) mouse is a mouse model that closely resembles human immunoglobulin A (IgA) nephropathy [17]. This disease is the most common glomerulonephritis worldwide and is an important cause of end-stage renal failure [18]. An abnormally increased level of serum IgA leads to formation of IgA-containing immune complexes, and their subsequent mesangial deposition results in inflammation and glomerular injury by complement 3 (C3) activation [18]. HIGA mice gradually exhibit these pathological findings, i.e., mesangial proliferation associated with IgA deposition in glomeruli, which becomes prominent at age of 25 weeks [17]. Mesangial lesions showed mild to moderate cell proliferation until 40 weeks of age, and moderate to marked mesangial matrix expansion was observed at age of more than 40 weeks [17].

Here, we identified the *Atractylodes* species present in a *Sojutsu* sample and extracted it to isolate pharmacologically active constituents. We then monitored their effects on NO production in interleukin (IL)-1β-treated hepatocytes. Subsequently, we administrated HIGA mice daily with the ethyl acetate-soluble fraction of the *Sojutsu* extract, which contained the hydrophobic constituents, to investigate whether this *Sojutsu* fraction improves mesangial lesions of IgA nephropathy in HIGA mice.

## Materials and methods

### General experimental procedures

Nuclear magnetic resonance (NMR) spectra were recorded using a JNM-ECS400 NMR spectrometer (JEOL Ltd., Tokyo, Japan) operated at 400 MHz (^1^H) with tetramethylsilane as internal standard. Gas chromatograph–mass spectrometry (GC–MS) analyses were performed using a Shimadzu QP 2010 mass spectrometer (Shimadzu, Kyoto, Japan) equipped with a Shimadzu GC2010 gas chromatography system and an AOC-20i autosampler. High-performance liquid chromatography (HPLC) analyses were performed using an LC-20AD pump equipped with an SPD-20A UV/VIS detector (Shimadzu Corporation). Column chromatography was run on silica gel (Silica gel 60; Nacalai Tesque Inc., Kyoto, Japan). Precoated thin-layer chromatography (TLC) was performed on silica gel 60 F_254_ plates (FUJIFILM Wako Pure Chemical Corporation, Osaka, Japan). Electron ionization–mass spectrometry (EI–MS) spectra were obtained with a JMS-700 MStation mass spectrometer (JEOL Ltd.). The optical rotations were measured on a DIP-1000 polarimeter (JASCO Corporation, Hachioji, Tokyo, Japan).

### Plant materials and reagents

An *Atractylodes* rhizome sample collected from Shaanxi Province, China was purchased from Tochimoto Tenkaido Co. Ltd and authenticated as *Sojutsu* by Dr. Yutaka Yamamoto (Tochimoto Tenkaido Co. Ltd., Osaka, Japan). This sample consisted of small cut-up pieces of rhizome. Whole-plant specimens of *A. lancea*, *A. chinensis*, and *A. japonica* were collected from the Herb Garden of Yokohama University of Pharmacy (Yokohama, Japan) and authenticated. These voucher samples were deposited in the Ritsumeikan Herbarium of Pharmacognosy, Ritsumeikan University, under code numbers RIN-AC-020 (*Sojutsu* sample), AC-021 (*A. chinensis*), AL-022 (*A. lancea*), and AJ-023 (*A. japonica*). As standards, β-eudesmol and (−)-α-bisabolol were purchased from FUJIFILM Wako Pure Chemical Corporation and Sigma-Aldrich Corp. (St. Louis, MO, USA), respectively.

### Species identification of *Atractylodes* samples

A *Sojutsu* sample or a leaf of *A. lancea*, *A. chinensis*, and *A. japonica* was separately pulverized, and genomic DNA was extracted using a NucleoSpin^®^ Plant II Kit (MACHEREY-NAGEL GmbH & Co. KG, Düren, Germany). The resultant DNA was subjected to polymerase chain reaction (PCR) to amplify the ITS1 located between the *18S rRNA* and *5.8S rRNA* genes using 5′-tgtaaaacgacggccagtAACGACCCGCGAACATGTAA-3′ and 5′-atttaggtgacactatagaCGAGAGTCGTTTGTGTTTCC-3′, where the sequences to which the pUC/M13-M4 primer and SP6 primer hybridize are underlined. The resultant DNA fragments were directly sequenced. The elongation factor-1α (*EF*) gene was amplified by PCR using 5′-ATTGGAGGTATCGGGACTGTACCTGTTGG-3′ and 5′-TGACCCGGATGGTTCATGATGATGACCTGAG-3′. The resultant DNA fragments were inserted into a pGEM-T Easy vector (Promega Corporation, Madison, WI, USA) and used to transform *Escherichia coli* strain DH5α. The nucleotide sequences were then confirmed. These sequences were deposited in the DNA Data Bank of Japan/European Bioinformatics Institute (DDBJ/EMBL)/GenBank under accession numbers LC465406–LC465417.

### Isolation of constituents from a *Sojutsu* sample

**Fig. 1:**
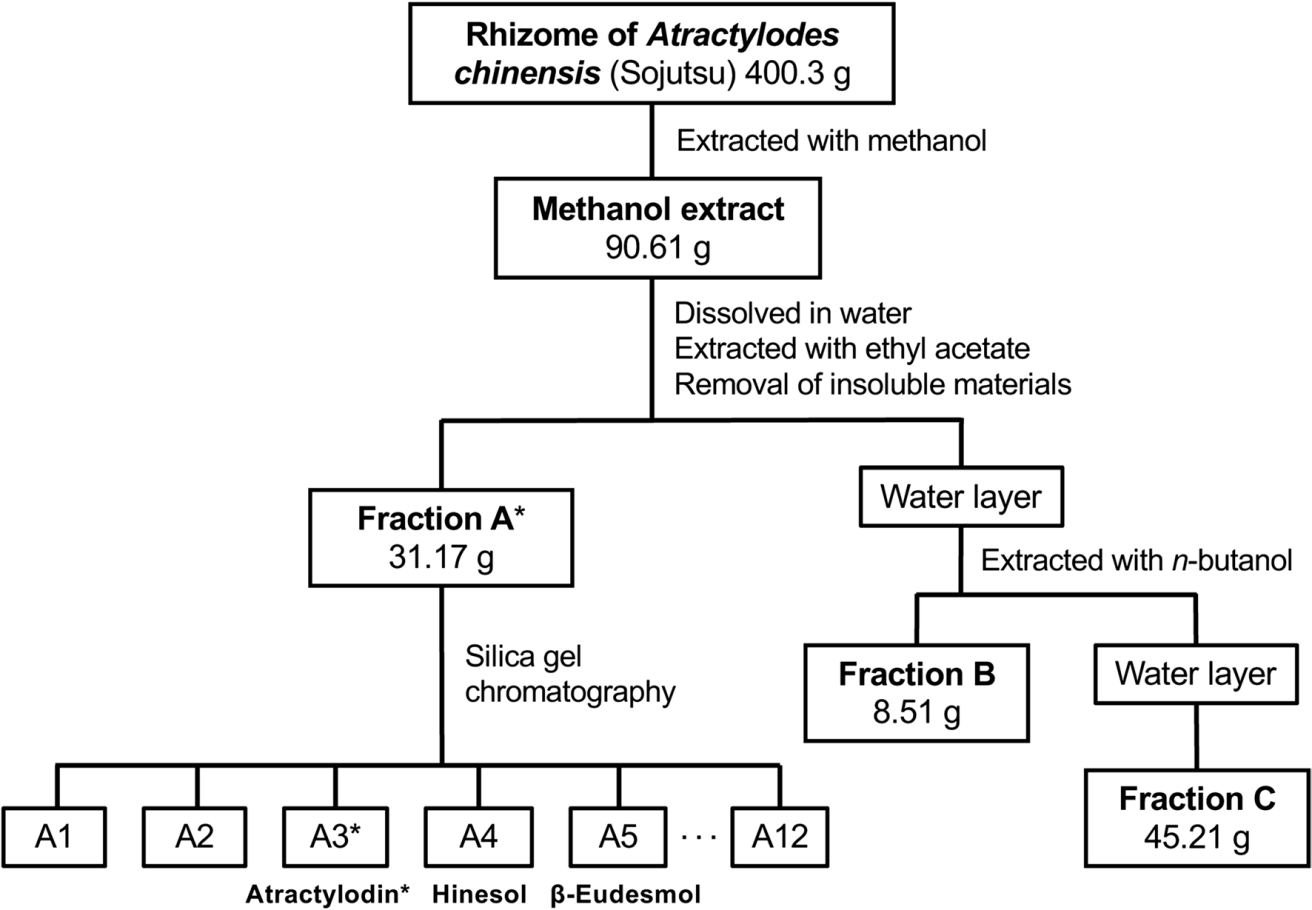
Purification of constituents from *A. chinensis* rhizome (*Sojutsu*). Flowchart of the procedures used to fractionate constituents from the *A. chinensis* rhizome. The plant material was extracted with methanol, and the dried extract was dissolved in water and sequentially fractionated with EtOAc (A), *n*-butanol (B), and water (C) by hydrophobicity. The weight of each crude fraction is indicated. Constituents are shown under the relevant subfraction. Asterisk: a fraction, subfraction, or constituent that markedly inhibited NO induction in this study

The *Sojutsu* sample (400.3 g), identified as *A. chinensis* rhizome (ACR), was pulverized, extracted by methanol under reflux, and fractionated, as previously described [14, 19]. Briefly, the resultant extract (90.61 g) was suspended in water and successively extracted with ethyl acetate (EtOAc) and *n*-butanol (Fig. 1). These layers were concentrated to prepare an EtOAc-soluble fraction (fraction A), an *n*-butanol-soluble fraction (fraction B), and a water-soluble fraction (fraction C). Fraction A, which showed NO-suppressing activity, was further purified by silica gel 60 column chromatography (5.0 cm i.d. × 23 cm) by elution with *n*-hexane:ethyl acetate (100:0 → 0:100) to yield 12 subfractions (A1–A12).

Subfraction A3 (201 mg) was crystallized from MeOH to afford pale-brown needles (25.6 mg), m.p. 48.5–50 °C. EI-MS *m*/*z* (%): 182 (M^+^, 100), 152(55), 139 (9.8); HR-EI-MS [M]^+^*m*/*z* 182.0725 (calculated for C_13_H_10_O: 182.0732); ^1^H NMR (400 MHz, CDCl_3_): *δ* 1.83 (1H, dd, *J* = 6.8, 1.6 Hz, H-9), 5.59 (1H, br d, *J* = 15.6 Hz, H-7), 6.10 (1H, d, *J* = 16.0 Hz, H-2), 6.33 (1H, dq, *J* = 15.6, 6.8 Hz, H-8), 6.37 (1H, d, *J* = 3.6 Hz, H-3′), 6.41 (1H, dd, *J* = 3.6, 1.6 Hz, H-4′), 6.79 (1H, d, *J* = 16.0 Hz, H-1), 7.38 (1H, d, *J* = 1.6 Hz, H-5′); ^13^C NMR (100 MHz, CDCl_3_): *δ* 19.0 (C-9), 72.6 (C-5), 77.2 (C-4), 80.2 (C-3), 81.9 (C-6), 104.8 (C-2), 109.9 (C-7), 110.1 (C-4′), 112.1 (C-3′), 130.7 (C-1), 143.5 (C-5′), 143.7 (C-8), 151.9 (C-2′). This compound was identified as atractylodin on the basis of mass and ^1^H NMR and ^13^C NMR spectral analysis and by comparison with published data [20].

Subfraction A4 (335 mg) was purified by silica gel column chromatography (1.0 cm i.d. × 10 cm) using *n*-hexane:ethyl acetate (95:5), followed by preparative TLC [*n*-hexane:CHCl_3_:MeOH (35:11:1)] to afford colorless needles (35.7 mg); m.p. 50–53 °C; [*α*]_D_^25^ +41.1° (*c* 0.795, CHCl_3_). EI–MS *m*/*z* (%): 222 (M^+^, 6.3), 204 (M^+^-H_2_O, 77), 189 (60), 161 (100); HR–EI–MS *m*/*z* 222.1966 [M]^+^ (calculated for C_15_H_26_O: 222.1984); ^1^H NMR (400 MHz, CDCl_3_): *δ* 0.92 (3H, d, *J* = 6.8 Hz, H-12), 1.19 and 1.21 (each 3H, s, H-14 and H-15), 1.34–1.62 (4H, m, H-3 and H-4), 1.37 (2H, m, H-9), 1.56 (1H, m, H-10), 1.68 (3H, dt, *J* = 1.2, 2.0 Hz, H-11), 1.70 (2H, m, H-1), 1.94 (3H, m, H-2 and H-8), 5.32 (1H, m, H-7); ^13^C NMR (100 MHz, CDCl_3_): *δ* 16.2 (C-12), 19.9 (C-11), 24.2 (C-8), 27.7 (C-4), 27.9 (C-3), 28.0 and 28.4 (C-14 and C-15), 33.2 (C-9), 35.7 (C-1), 36.7 (C-10), 48.4 (C-5), 51.4 (C-2), 72.0 (C-13), 121.7 (C-7), 140.1 (C-6). The assignments of these signals were based on the H–H correlation spectroscopy (COSY), heteronuclear multiple-quantum coherence (HMQC), and heteronuclear multiple-bond correlation (HMBC) spectra. The MS, ^1^H NMR, and ^13^C NMR spectral data were in agreement with reported data [4, 21] for (−)-hinesol. But the [*α*]_D_ (+41.1° in CHCl_3_) of this compound was opposite to the literature value (−40° in CHCl_3_) of (−)-hinesol [22] and agreed with the literature value (+35.4° in CHCl_3_) of the synthesized (+)-hinesol [23]. Therefore, this compound was determined as (+)-hinesol. (+)-Hinesol was isolated from a natural source for the first time.

Subfraction A5 (886 mg) was purified by silica gel column chromatography (1.5 cm i.d. × 20 cm) using *n*-hexane:ethyl acetate (95:5), followed by preparative TLC [*n*-hexane:CHCl_3_:MeOH (35:10:5)] to afford colorless needles (30.2 mg); m.p. 74.5–76 °C; [*α*]_D_^21^ −55.3° (*c* 0.635, CHCl_3_). EI–MS *m*/*z* (%): 222 (M^+^, 8.4), 204 (15), 189 (19), 164 (75), 149 (100); HR–EI–MS *m*/*z* 222.1970 [M]^+^ (calculated for C_15_H_26_O: 222.1984); ^1^H NMR (400 MHz, CDCl_3_): *δ* 0.72 (3H, s, H-14), 1.14 (1H, d, *J* = 12.8) and 1.60 (1H, m) (H-6), 1.21 (6H, s, H-12 and H-13), 1.21 and 1.44 (each 1H, m, H-1), 1.23–1.31 (2H, m, H-8), 1.29 and 1.53 (each 1H, m, H-9), 1.36 (1H, m, H-7), 1.44 and 1.60 (each 1H, m, H-2), 1.77 (1H, m, H-5), 2.00 and 2.31 (each 1H, m, H-3), 4.45 and 4.72 (each 1H, q, *J* = 1.6 Hz, H-15); ^13^C-NMR (100 MHz, CDCl_3_): *δ* 16.3 (C-14), 22.4 (C-8), 23.5 (C-2), 25.0 (C-6), 27.1 and 27.2 (C-12 and C-13), 35.9 (C-10), 36.9 (C-3), 41.1 (C-9), 41.8 (C-1), 49.4 (C-7), 72.9 (C-11), 105.3 (C-15), 151.2 (C-4). This compound was identified as β-eudesmol on the basis of [*α*]_D_, mass, ^1^H NMR, and ^13^C NMR spectral analysis and by comparison with published data [24].

### GC–MS analyses of atractylodin, hinesol, β-eudesmol, and α-bisabolol

Atractylodin, hinesol, β-eudesmol, and α-bisabolol were analyzed by GC–MS using a DB-5MS capillary column (0.25 mm × 30 m, 0.25 μm film thickness, Agilent Technologies, Santa Clara, CA, USA). The analytical conditions were as follows: ionization mode EI, injector and transfer line temperature, 200 °C; oven temperature programmed from 50 to 300 °C at 10 °C/min; carrier gas, helium (1 mL/min); splitless mode; ionization voltage, 70 eV; ionization current, 300 μA. Commercially purchased (−)-α-bisabolol was used as standard. Atractylodin, hinesol, β-eudesmol, and α-bisabolol were accurately weighed and dissolved in MeOH to make stock solutions of 1.0 mg/mL. Then, series of hinesol and β-eudesmol standard solutions (0.025, 0.05, and 0.1 μg/mL) were prepared by diluting the stock solutions to make calibration curves. Series of atractylodin and α-bisabolol standard solutions (0.01, 0.02, and 0.04 μg/mL) were similarly prepared. A calibration curve of each standard compound was calculated by plotting peak areas (*y*) against a series of injection amounts (*x*, μg). The calibration equation and correlation coefficient of three standard compounds were as follows; atractylodin, *y* = 345,556,992*x* + 263,875 (*R*^2^ = 1.000); hinesol, *y* = 175,938,000*x* + 3,302,345 (*R*^2^ = 0.991); β-eudesmol, *y* = 197,417,365*x* + 6,056,334 (*R*^2^ = 0.998). Fraction A was accurately weighed and dissolved in MeOH to make a sample solution of 200 μg/mL. The sample solution (1.0 μL) was analyzed in duplicate. The peak areas of atractylodin, hinesol, and β-eudesmol in the sample solution were fit to the calibration curves, and the amounts of atractylodin, hinesol, and β-eudesmol in 1.0 μL of sample solution calculated. The amounts of atractylodin, hinesol, and β-eudesmol in 1.0 μL of sample solution (0.2 μg of fraction A) were calculated to be 0.0098 μg, 0.0490 μg, and 0.0459 μg, respectively. Therefore, the contents of atractylodin, hinesol, and β-eudesmol in fraction A were 4.88%, 24.5%, and 23.0%, respectively. α-Bisabolol was not quantified because it was present in trace amount, but its presence was confirmed by GC–MS. The fragment pattern of the peak at *t*_R_ 17.9 min was consistent with that of α-bisabolol (data not shown).

### Animal experiments

All animal care and experimental procedures were performed in accordance with the laws and guidelines of the Japanese government and were approved by the Animal Care Committee of Ritsumeikan University, Biwako-Kusatsu Campus. Specific pathogen-free (SPF), male Wistar rats (5–6 weeks old; Charles River Laboratories Japan, Inc., Yokohama, Japan) were housed at 21–23 °C under a 12 h light–dark cycle and fed with a CRF-1 diet (γ-ray-irradiated; Charles River Laboratories Japan) and had water available ad libitum. Before preparation of hepatocytes, the animals were acclimated to their housing for a week.

Female HIGA mice and female BALB/c mice (SPF, 4 weeks old; Japan SLC, Inc., Hamamatsu, Japan) were fed daily with a CRF-1 diet with water available ad libitum. BALB/c mice were used as healthy controls. These mice were fed with a CRF-1 diet with or without 1% (w/w) fraction A of ACR extract from 10 weeks until 30 weeks of age (*n* = 3–4 per group). At age of 20 and 30 weeks, three mice in each group were euthanized by cervical dislocation, and blood was drawn from the heart to obtain serum. Perirenal and parametrial white adipose tissue was excised and weighed. The kidneys were excised and soaked in RNA*later* stabilization solution (Thermo Fisher Scientific Inc., Carlsbad, CA, USA) for subsequent RNA preparation and histochemistry.

### Primary cultured rat hepatocytes

Hepatocytes were isolated from livers of male Wistar rats (SPF, 250–300 g body weight), according to a previously published method [15, 25]. Briefly, the liver was perfused with collagenase, and the dispersed cells were centrifuged, resuspended, and seeded at 1.2 × 10^6^ cells per 35-mm-diameter dish. The cells were subsequently incubated at 37 °C for 2 h, and the medium was replaced with fresh medium. The cells were incubated at 37 °C overnight until analysis on day 1.

### Estimation of NO production and lactate dehydrogenase (LDH) activity

Each fraction or constituent was added to the medium on day 1, and the hepatocytes were incubated for 8 h. Nitrite (a stable metabolite of NO) in the medium was measured using the Griess method to measure NO levels [26, 27]. The NO levels in the presence and absence of recombinant rat IL-1β (≥ 98% purity; PeproTech, Rocky Hill, NJ, USA) in the medium were set at 100% and 0%, respectively, in all the assays. The half-maximal inhibitory concentration (IC_50_) value against nitrite was determined in triplicate for at least three different concentrations of an extract or constituent [27]. An IC_50_ value of an extract or constituent was calculated to determine its ability to suppress NO production, unless the compound was cytotoxic. The LDH activity in the medium was measured using an LDH cytotoxicity detection kit (Takara Bio Inc., Otsu, Japan) to estimate cytotoxicity.

### Direct NO quenching activity

Each constituent was added to a medium containing 25 μM NaNO_2_ and incubated at 37 °C for 1.5 h [27]. This medium was then mixed with Griess reagent and incubated at room temperature for 5 min. The absorbance at 540 nm was measured in triplicate to measure the reduction of nitrite mediated by the constituent. The nitrite level in the medium containing NaNO_2_ alone was set at 100%.

### Western blot analysis

Western blotting was performed, as previously described [14]. Briefly, hepatocytes were treated with 1 nM IL-1β and a fraction or constituent for 8 h and lysed in the presence of protease inhibitor cocktail (Nacalai Tesque, Inc.). The resultant lysates were run on a 10% sodium dodecyl sulfate–polyacrylamide gel and blotted onto a Sequi-Blot membrane (Bio-Rad, Hercules, CA, USA). After blocking with 5% skimmed milk, immunostaining was performed using primary antibodies against iNOS (Clone 54; BD Biosciences, San Jose, CA, USA) and β-tubulin (internal control; Cell Signaling Technology Inc., Danvers, MA, USA) and then horseradish peroxidase (HRP)-conjugated anti-immunoglobulin Fc antibody. The protein was visualized using enhanced chemiluminescence blotting detection reagents (GE Healthcare Biosciences Corp., Piscataway, NJ, USA) and detected using an Amersham Imager 600 (GE Healthcare).

### Quantitative reverse transcription-polymerase chain reaction (qRT-PCR)

Total RNA was prepared from rat hepatocytes and the kidney of each mouse (in RNA*later* Solution) using Sepasol I Super G solution (Nacalai Tesque, Inc.) and purified with the RNAqueous kit and the TURBO DNA-free kit (Applied Biosystems) [15, 16]. The cDNA was reverse-transcribed from total RNA and amplified by PCR using the primers presented in Table 1 and in Ref. [16]. The mRNA levels were quantitatively measured in triplicate by real-time PCR using SYBR Green I and the Thermal Cycler Dice real-time system (Takara Bio Inc.), and the values were normalized to *EF* mRNA [16].

**Table 1:** Primers used for quantitative RT-PCR to detect mRNA

mRNA (species)	Sequence (5′ → 3′)	Direction	cDNA (bp)
iNOS (rat)	CCAACCTGCAGGTCTTCGATG	Forward	254
	GTCGATGCACAACTGGGTGAAC	Reverse	
iNOS (mouse)	TTCAACACCAAGGTTGTCTGCA	Forward	179
	AACACAGCATACCTGAAGGTGT	Reverse	
TNF-α (rat)	TCCCAACAAGGAGGAGAAGTTCC	Forward	275
	GGCAGCCTTGTCCCTTGAAGAGA	Reverse	
CCL2 (rat)	GCTGTCTCAGCCAGATGCAGTTA	Forward	223
	GATCTCACTTGGTTCTGGTCCAG	Reverse	
LCN2 (rat)	TCACCCTGTACGGAAGAACCAAG	Forward	126
	CAATGCATTGGTCGGTGGGAACA	Reverse	
LCN2 (mouse)	GAAATATGCACAGGTATCCTCAGG	Forward	144
	CCTTGGTTCTTCCATACAGGGTAA	Reverse	
EF mRNA (mouse, rat)	TCTGGTTGGAATGGTGACAACATGC	Forward	335
	CCAGGAAGAGCTTCACTCAAAGCTT	Reverse	

*RT-PCR* reverse-transcription polymerase chain reaction, *bp* base pairs, *iNOS* inducible nitric oxide synthase, *TNF-α* tumor necrosis factor α, *CCL2* chemokine C–C motif ligand 2, *LCN2* lipocalin 2, *EF* elongation factor 1α

### Examination of serum

Serum was obtained from blood drawn from mouse hearts, and the levels of blood urea nitrogen (BUN) and creatinine were measured. The serum levels of LCN2 and G-CSF were determined by enzyme-linked immunosorbent assay (ELISA) using a Mouse NGAL ELISA kit (R&D Systems, Minneapolis, MN, USA) and a Mouse G-CSF Quantikine ELISA kit (BioPorto Diagnostics A/S, Hellerup, Denmark).

### Immunohistochemistry

Kidney specimens taken from 30-week-old mice (in RNA*later* solution) were fixed in 4% formaldehyde and embedded in paraffin. Sections (3–5 mm) were deparaffinized and stained with hematoxylin and eosin. To detect IgA deposition in the glomeruli, the deparaffinized sections were treated with 3% hydrogen peroxide, blocked with 10% normal goat serum (Nichirei Bioscience, Tokyo, Japan), and incubated with a goat anti-mouse IgA antibody conjugated with HRP (Bethyl Laboratories, Montgomery, TX, USA; 200:1 dilution) at 23 °C for 2 h. Immunoreactivity was detected with 3,3′-diaminobenzidine tetrachloride (Nichirei Bioscience) as substrate. The resultant sections were counterstained with hematoxylin and examined under a BA210EINT research biological digital microscope (Shimadzu Rika, Tokyo, Japan). The glomeruli showing immunoreactivity to IgA were quantified in a blinded manner, and 10 low-power fields (LPFs; 100×) were counted five times.

### Statistical analysis

The results using hepatocytes are representative of at least three independent experiments that yielded similar findings. The values are presented as mean ± standard deviation (SD). The differences were analyzed using Student’s *t* test. Significance was set at *P* < 0.05 and *P* < 0.01.

## Results

### Identification of *Atractylodes* species in a *Sojutsu* sample

The *Atractylodes* species present in the crude *Sojutsu* drug used in this study was unknown; therefore, the genomic DNA of the *Sojutsu* was subjected to PCR and subsequent DNA analyses. As references, we used leaves of *A. lancea*, *A. chinensis*, and *A. japonica* that were botanically authenticated. The ITS1 sequence between the *18S*–*5.8S rRNA* genes [2, 3] derived from the *Sojutsu* samples was compared with those obtained from the leaves. The 167-base-pair (bp) ITS1 sequence from five pieces of the *Sojutsu* sample was identical to those of *A. chinensis* leaf, i.e., 100% homology, whereas it was 99.4% (166 bp/167 bp) and 97.0% (162 bp/167 bp) identical to those of the *A. lancea* and *A. japonica* leaves, respectively. These data indicate that the *Sojutsu* sample used in this study was an *A. chinensis* rhizome. To avoid confusion, we hereafter call this *Sojutsu* sample “*Atractylodes chinensis* rhizome,” instead of “*Atractylodes lancea* rhizome,” as defined by the Japanese Pharmacopoeia [1].

The *EF* gene of the *Sojutsu* sample was also examined by sequencing because *EF* is a ubiquitously expressed housekeeping gene. The 230-bp *EF* sequence of the *Sojutsu* sample was identical to those of the *A. lancea* and *A. japonica* leaf samples. These data indicate that the *EF* gene sequences are highly conserved among *Atractylodes* species and cannot be used to discriminate *Atractylodes* species.

### Suppression of NO production by fractions from ACR extract

The ACR was extracted with methanol, according to a previously published method [14, 19]. The resultant ACR extract was sequentially fractionated into three fractions based on hydrophobicity using EtOAc (fraction A), *n*-butanol (fraction B), and water (fraction C) (Fig. 1).

**Fig. 2:**
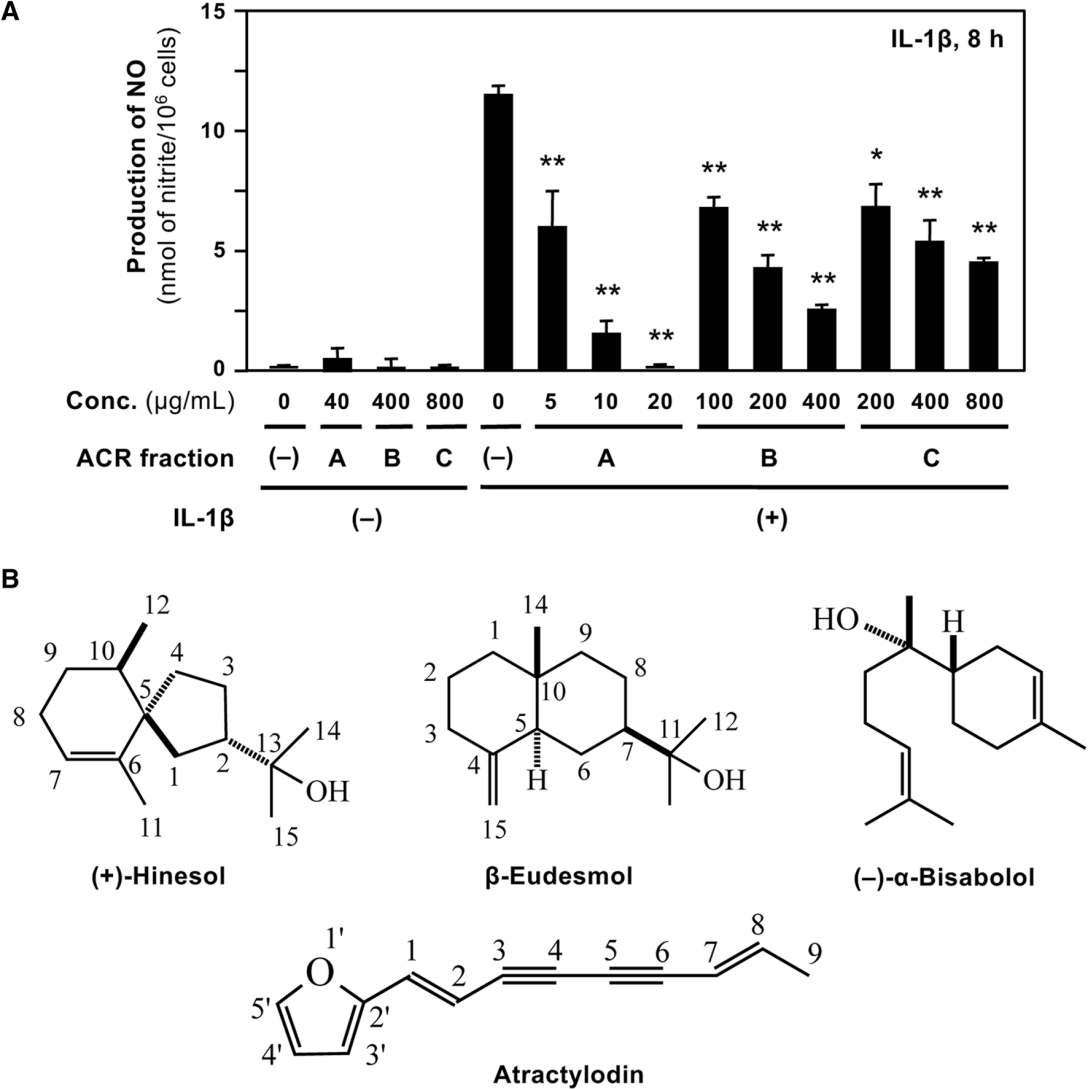
Fraction A of *A. chinensis* rhizome extract suppresses NO production. **a** The effects of *A. chinensis* rhizome (ACR) fractions on NO production. Hepatocytes were treated with 1 nM IL-1β in presence or absence of each ACR fraction (A–C) for 8 h. The levels of nitrite (a major metabolite of NO) were measured in the culture medium. Data are mean ± standard deviation (SD) of triplicate experiments. *P < 0.05 and **P < 0.01 versus IL-1β alone. **b** Chemical structures of the principal constituents in the rhizome of *A. chinensis*. Atractylodin, β-eudesmol, hinesol, and (−)-α-bisabolol are depicted

Because iNOS expression and NO production are induced in rat hepatocytes by the proinflammatory cytokine IL-1β [12], we monitored the NO-suppressing activity of the ACR extract and its constituents. All the fractions inhibited NO induction in IL-1β-treated hepatocytes in a dose-dependent manner (Fig. 2a). The LDH activity of the medium indicated that none of the fractions produced cytotoxicity at the concentrations indicated (data not shown). Among the three ACR extract fractions, fraction A markedly suppressed NO production with IC_50_ value of 12.3 μg/mL (Table 2) and decreased the level of iNOS protein in hepatocytes (data not shown). Fraction A, i.e., the EtOAc-soluble fraction, was expected to include hydrophobic constituents, which may suppress IL-1β-induced NO production.

**Table 2:** Fractionation of *A. chinensis* rhizome extract and its effects on nitric oxide production

Fraction	Yield (%)^a^	IC_50_ (μg/mL)^b^	Constituent	Content (%)^c^	IC_50_ (μM)^b^
Methanol extract	100	–	–		
A (ethyl-acetate-soluble)	36.7	12.3 ± 2.77	–	100	
			(+)-Hinesol	23.0	NA
			β-Eudesmol	24.5	NA
			(−)-α-Bisabolol	ND	254.0 ± 18.5
			Atractylodin	4.88	8.25 ± 1.73
B (*n*-butanol-soluble)	10.0	197 ± 60.4	–	–	
C (water-soluble)	53.3	NA	–	–	

*NA* not applied due to low activity, *ND* not determined

^a^Percentage calculated as the weight of each fraction divided by the sum of three fractions

^b^The half-maximal inhibitory concentration of nitric oxide (NO) production in IL-1β-treated hepatocytes (mean ± standard deviation). At least three experiments were performed to determine these values

^c^The content of each constituent was measured by GC–MS and is shown as a percentage of the dry weight of fraction A

### Suppression of NO production by ACR fraction A constituents

Constituents were purified from the NO-suppressing fraction A of ACR extract. From subfractions A3 to A5, atractylodin, β-eudesmol, and hinesol were isolated (Fig. 1). Gas chromatography–mass spectrometry (GC–MS) analysis was performed to estimate the content of each constituent isolated from ACR fraction A. β-Eudesmol and hinesol were the major constituents in fraction A (Table 2). Traces of α-bisabolol were also detected by GC–MS; however, its content could not be measured.

Next, we examined whether these constituents in ACR fraction A could suppress NO induction in IL-1β-treated hepatocytes (Table 2). Among the constituents, atractylodin exhibited high potency to suppress NO production, with IC_50_ value of 8.25 μM. Atractylodin did not show cytotoxicity at concentrations up to 100 μM (data not shown). When the NO-quenching activity of atractylodin was measured, no significant changes in NO levels were observed compared with the NO levels in the medium containing NaNO_2_ alone (data not shown), indicating that atractylodin did not directly quench NO. Therefore, we used atractylodin for the subsequent experiments.

### Inhibition of iNOS gene expression in hepatocytes by atractylodin

**Fig. 3:**
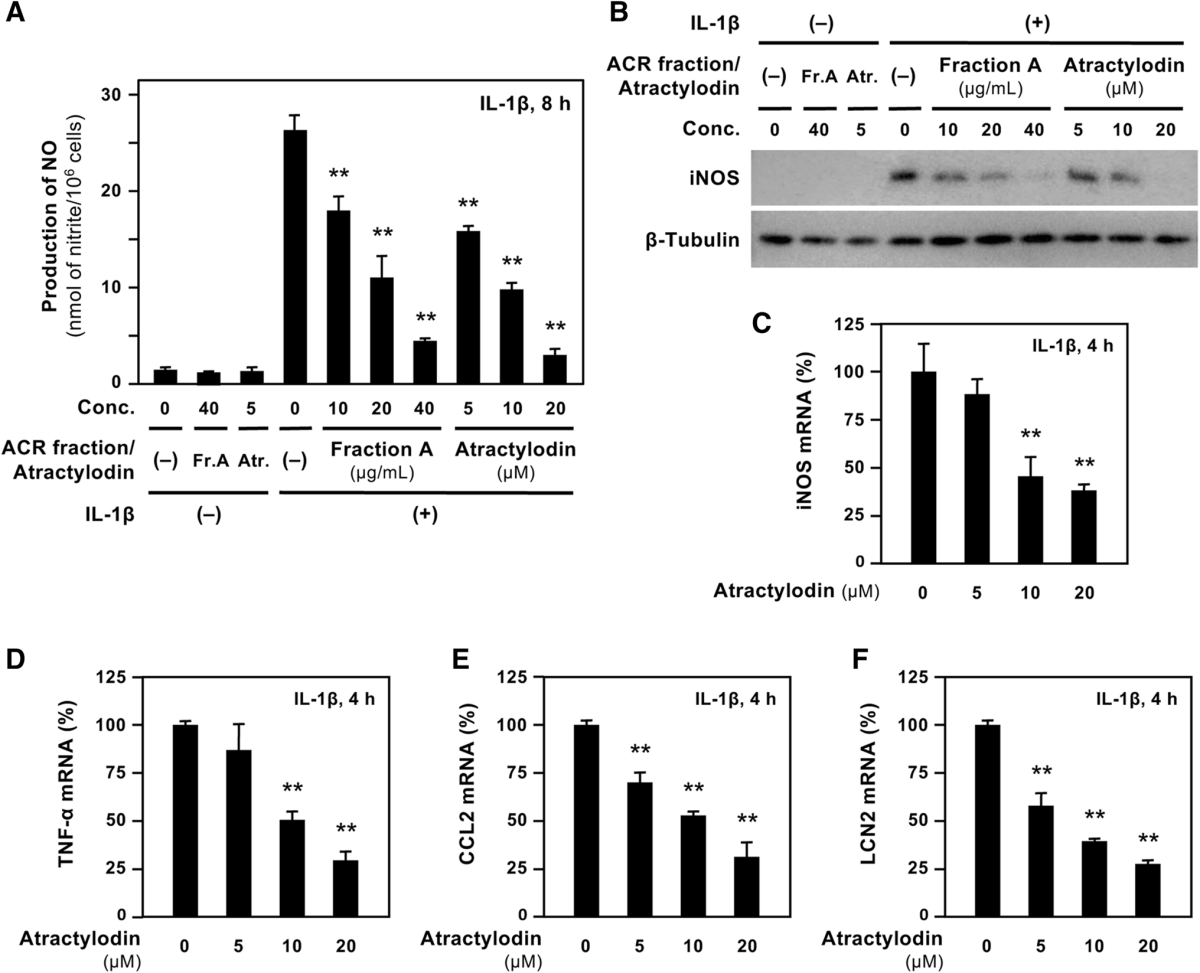
Atractylodin suppresses IL-1β-induced *iNOS* gene expression in hepatocytes. **a** The effects of atractylodin on NO production. Hepatocytes were treated with 1 nM IL-1β in absence or presence of fraction A of *A. chinensis* rhizome (ACR) extract, or atractylodin for 8 h. The nitrite levels were then measured in the medium; data presented as mean ± SD (*n* = 3). **P < 0.01 versus IL-1β alone. **b** The effects of atractylodin on iNOS protein levels. Hepatocytes were treated similarly to **a** and analyzed by Western blotting. The cell extracts (20 μg per lane) were used to detect iNOS (130 kDa) and β-tubulin (55 kDa; internal control). **c** The effects of atractylodin on *iNOS* mRNA levels in hepatocytes. After incubation with 1 nM IL-1β and atractylodin for 4 h, total RNA was prepared, then quantitative reverse transcription-polymerase chain reaction (qRT-PCR) using the primers presented in Table 1 was performed to detect *iNOS* mRNA using elongation factor-1α (*EF*) mRNA as internal control. No amplification was observed when cells were treated with atractylodin alone (data not shown). The levels of *iNOS* mRNA were normalized to those of *EF* mRNA and are expressed as percentages (mean ± SD, *n* = 3). **P < 0.01 versus IL-1β alone. **d**–**f** The effects of atractylodin on the expression of various mRNAs in hepatocytes. Similar to **c**, total RNA was analyzed by qRT-PCR to detect tumor necrosis factor α (*TNFα*; **d**), chemokine C–C motif ligand 2 (*CCL2*; **e**), and lipocalin 2 (*LCN2*; **f**). The normalized mRNA data (mean ± SD, *n* = 3) are expressed as percentages. **P < 0.01 versus IL-1β alone

The effects of atractylodin in ACR extract fraction A on *iNOS* gene expression were further examined. When atractylodin was added with IL-1β to the hepatocyte culture medium, it inhibited the induction of NO and the iNOS protein (Fig. 3a, b). qRT-PCR analysis revealed that atractylodin reduced *iNOS* mRNA levels in a dose-dependent fashion (Fig. 3c). Atractylodin also decreased the levels of the iNOS antisense transcript, which stabilizes the *iNOS* mRNA [28] (data not shown). Together, these data imply that atractylodin in ACR fraction A downregulates *iNOS* gene expression by reducing *iNOS* mRNA levels.

### Inhibition of proinflammatory cytokine mRNAs in hepatocytes by atractylodin

When an antiinflammatory agent suppresses NO production, the expression of genes involved in inflammation are inhibited [16, 29]. Therefore, we examined whether atractylodin inhibited the expression of tumor necrosis factor α (*TNFα*), chemokine C–C motif ligand 2 (*CCL2*), and lipocalin 2 (*LCN2*) mRNAs in IL-1β-treated hepatocytes (Fig. 3d–f). LCN2 is released by various cell types and is a biomarker of inflammation and infection [30]. While IL-1β increased the levels of *TNFα*, *CCL2*, and *LCN2* mRNAs, atractylodin decreased the levels of these mRNAs. Together, these findings indicate that atractylodin has antiinflammatory effects and that fraction A of the ACR extract may possess antiinflammatory effects.

### Effects of ACR fraction A on body weight and adipose tissue of HIGA mice

To investigate the effects of *Sojutsu* on the kidney, fraction A of the ACR extract was orally administrated to female HIGA mice, an IgA nephropathy model [17]. Because the pathological changes in renal glomeruli become apparent at age of 25 weeks [17, 31], HIGA mice were administrated ACR fraction A in a standard diet every day from the age of 10 to 30 weeks (for 20 weeks in total).

**Fig. 4:**
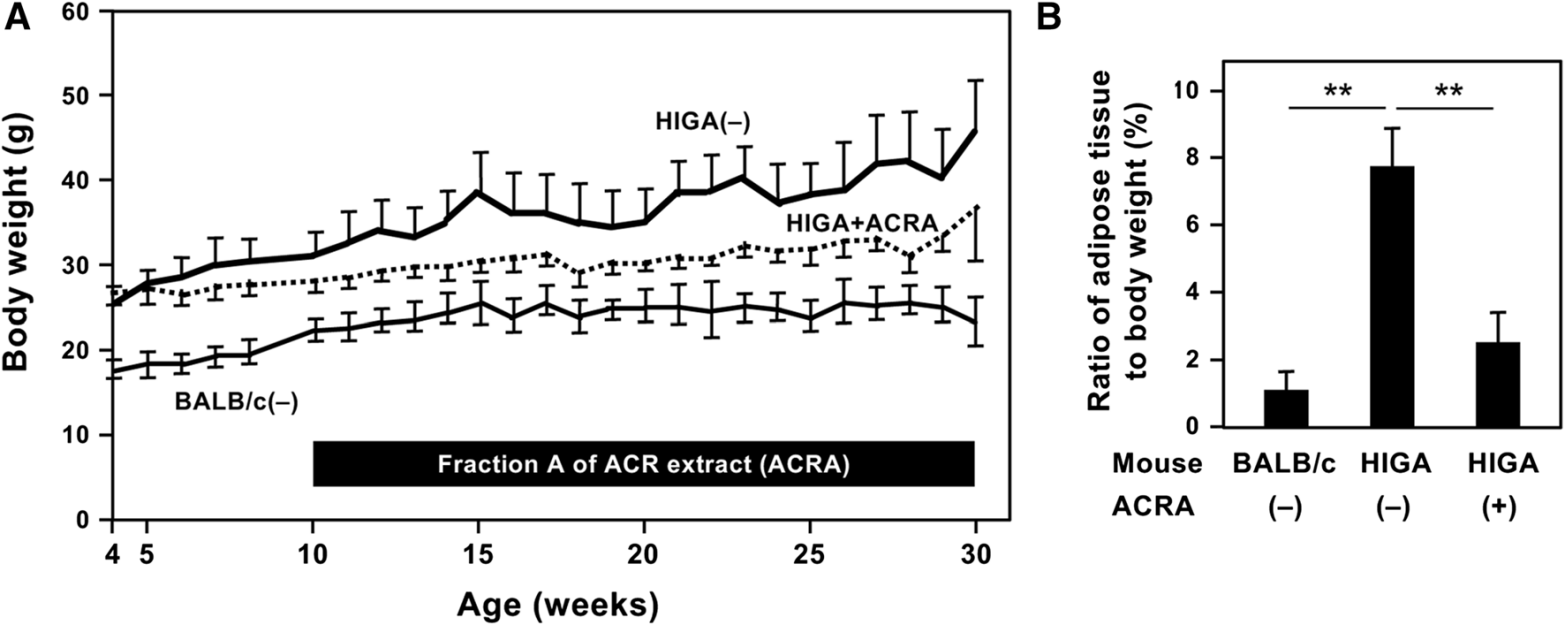
Fraction A of *A. chinensis* rhizome extract decreases the body weight and the adipose tissue weight of high immunoglobulin A (HIGA) mice. **a** The effects of fraction A of *A. chinensis* rhizome extract on the body weight of mice. Female HIGA mice were fed with a CRF-1 diet with 1% (w/w) fraction A of *A. chinensis* rhizome (ACRA) extract from 10 weeks until 30 weeks of age (HIGA + ACRA). As controls, female HIGA mice and BALB/c mice were fed a CRF-1 diet alone [HIGA(−) and BALB/c(−), respectively]. The body weight of each group is shown as the mean ± SD. BALB/c(−), thin line; HIGA(−), thick solid line; and HIGA + ACRA, broken line. **b** The effects of fraction A of *A. chinensis* rhizome extract on the weight of white adipose tissue. At the age of 30 weeks, mice were euthanized, and perirenal and parametrial white adipose tissue was excised from each mouse. The weight of white adipose tissue in each group (*n* = 3–4) is expressed as a percentage of total body weight (mean ± SD). **P < 0.01

First, the body weight of these mice was examined (Fig. 4a). The HIGA mice fed a standard diet alone [HIGA(−) mice; negative controls] were significantly heavier than BALB/c mice fed a standard diet alone [BALB/c(−) mice; healthy controls] (Fig. 4a). The body weight of HIGA mice administered ACR fraction A (HIGA + ACRA mice) was significantly lower than that of HIGA(−) mice during most of the administration period. The food intake of mice was almost the same among these three groups (i.e., 5.0–7.5 g per day per mouse), and marked differences of water consumption were not observed among these three groups (data not shown). Administration of ACR fraction A may therefore decrease the body weight of HIGA mice.

Next, the weight of white adipose tissue was measured at the age of 30 weeks. As shown in Fig. 4b, the percentage of white adipose tissue weight to body weight of HIGA + ACRA mice was significantly lower than that of the HIGA(−) mice. These results indicate that administration of ACR fraction A reduces the body weight increase of HIGA mice by reducing white adipose tissue.

### Decreased mesangial lesions in HIGA mice administered ACR fraction A

**Fig. 5:**
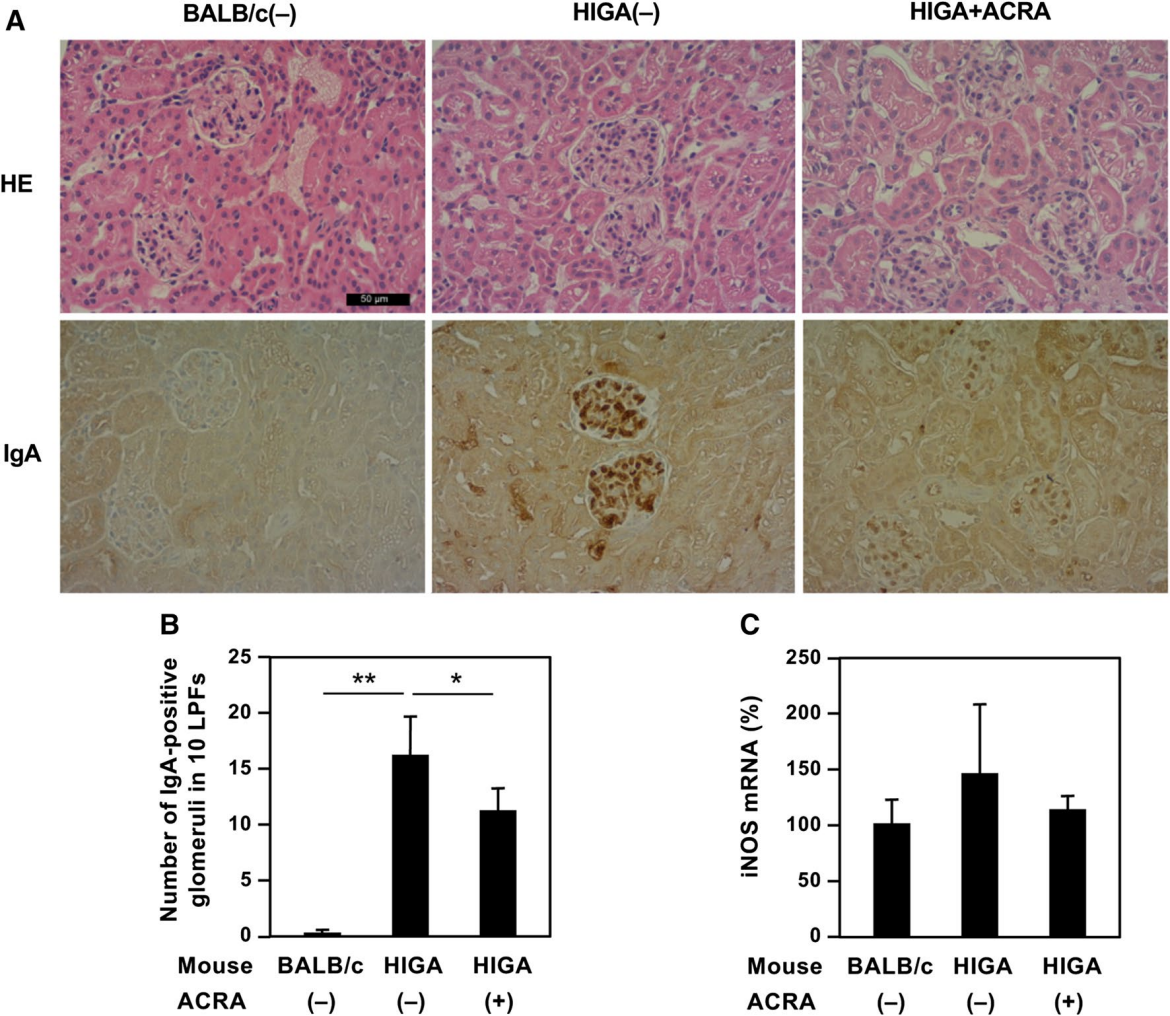
Fraction A of *A. chinensis* rhizome extract improves kidney pathology. **a** Pathological findings in the kidney. HIGA mice were orally administered fraction A of *A. chinensis* rhizome extract (ACRA) for 20 weeks (HIGA + ACRA). As controls, HIGA mice and BALB/c mice were fed a standard diet alone [HIGA(−) and BALB/c(−), respectively]. The kidneys of these mice at the age of 30 weeks were histologically examined. Kidney sections were subjected to hematoxylin and eosin (HE) staining and immunohistochemistry with an anti-immunoglobulin A antibody (IgA). Original magnification, ×400. Scale bar, 50 μm. **b** The effects of ACRA on the number of IgA-positive glomeruli. The number of glomeruli showing immunoreactivity to IgA was counted in 10 low-power fields (LPFs; ×100) five times. The number of these IgA-positive glomeruli with respect to the total number of glomeruli in 10 LPFs represents the mean ± SD. *P < 0.05 of HIGA + ACRA versus HIGA(−) group. **c** The effects of ACRA on the expression of *iNOS* mRNA in the kidney. Total RNA was extracted from each mouse and analyzed with quantitative RT-PCR using the primers presented in Table 1 to detect *iNOS* mRNA. The *iNOS* mRNA levels were normalized against the levels of elongation factor 1α (*EF*) mRNA and are represented as percentages (mean ± SD, *n* = 3–4 mice)

We examined the histology of glomeruli in the kidney. As shown in Fig. 5a, hematoxylin and eosin (HE) staining showed prominent mesangial proliferation in the glomeruli of HIGA(−) mice at the age of 30 weeks, compared with that in BALB/c(−) mice. In contrast, mesangial proliferation was decreased in the glomeruli of HIGA + ACRA mice.

Anti-IgA immunohistochemistry showed strong IgA staining in the mesangial cells of glomeruli in HIGA(−) mice. IgA-positive glomeruli were 82.0% of the total number of glomeruli. In contrast, weaker immunoreactivity to IgA was detected in the glomerular mesangial matrix of HIGA + ACRA mice, and the number of IgA-positive glomeruli was 57.0%. As shown in Fig. 5b, the number of IgA-positive glomeruli counted in 10 low-power fields in HIGA + ACRA mice was significantly lower than that in HIGA(−) mice. These data indicate that ACR fraction A decreased mesangial proliferation associated with IgA deposition.

Kidney function was assessed by analyzing serum samples of 20- and 30-week-old mice (Table 3). BUN levels of HIGA(−) mice were significantly higher at 20 weeks of age and lower at 30 weeks of age than those of BALB/c(−) mice. In addition, there were no significant differences in serum creatinine levels between the three groups.

**Table 3:** Examination of serum of mice fed with fraction A of *A. chinensis* extract

	Mouse	ACRA	Age	
			20 weeks	30 weeks
BUN (mg/dL)^a^	BALB/c	(−)	14.5 ± 2.4	23.5 ± 2.5
	HIGA	(−)	22.7 ± 1.6**	14.6 ± 4.2*
	HIGA	(+)	16.7 ± 1.9^##^	29.3 ± 6.5^#^
Creatinine (mg/dL)^a^	BALB/c	(−)	0.190^b^	0.193 ± 0.012
	HIGA	(−)	0.215 ± 0.039	0.163 ± 0.057
	HIGA	(+)	0.180 ± 0.000	0.230^b^
LCN2 (ng/mL)^a^	BALB/c	(−)	94.7 ± 13.8	66.6 ± 10.6
	HIGA	(−)	90.8 ± 21.9	81.2 ± 14.2
	HIGA	(+)	65.5 ± 7.5	72.6 ± 14.5
G-CSF (ng/mL)^a^	BALB/c	(−)	4.67 ± 2.14	3.11 ± 2.42
	HIGA	(−)	2.48 ± 2.30	10.81 ± 3.52*
	HIGA	(+)	6.54 ± 4.37	4.97 ± 3.01^#^

High immunoglobulin A (HIGA) mice and BALB/c (negative controls) mice were fed with a diet alone or a diet including 1%(w/w) fraction A of *A. chinensis* rhizome extract (ACRA) from 10 to 30 weeks of age

*P < 0.05 and **P < 0.01 versus BALB/c (−) mice. ^#^*P* < 0.05 and ^##^*P* < 0.01 versus HIGA (−) mice. Comparison performed between mice at the same age

^a^Blood was drawn from the heart, and the serum was subjected to analyses of blood urea nitrogen (BUN), creatinine, lipocalin-2 (LCN2), and granulocyte colony-stimulating factor (G-CSF). Values are mean ± standard deviation (*n* = 3–4). Serum levels of LCN2 and G-CSF were determined by enzyme-linked immunosorbent assay (ELISA)

^b^The mean of the measured values for two samples is shown, because one sample among three samples of a group could not be used due to hemolysis

### Other markers in HIGA mice administered the EtOAc-soluble ACR fraction

Finally, total RNA was prepared from mouse kidneys, and mRNA levels were assessed by qRT-PCR. The *iNOS* gene is thought to be expressed in macrophages in the kidney. Although renal *iNOS* mRNA levels in HIGA(−) mice tended to be higher than those in BALB/c(−) and HIGA + ACRA mice, these differences were not significant (Fig. 5c).

LCN2 is known as a biomarker of kidney damage [30]; however, no significant differences in renal *LCN2* mRNA levels were observed among these groups (data not shown). ELISA of mouse serum indicated that LCN2 levels in the HIGA + ACRA group were slightly lower than those of the HIGA(−) group, but these differences were not significant (Table 3).

Granulocyte colony-stimulating factor (G-CSF; also known as colony stimulating factor 3) is produced from monocytes and macrophages during inflammation [32], and their infiltration is observed in human glomerulonephritis [33]. Serum G-CSF levels in HIGA(−) mice were significantly higher than those in BALB/c(−) and HIGA + ACRA mice (Table 3), indicating that ACR fraction A reduces G-CSF expression in HIGA mice.

## Discussion

In this study, a *Sojutsu* sample defined by the Japanese Pharmacopoeia [1] was identified as the rhizome of *A. chinensis* by genomic DNA sequencing of *rRNA* genes. When an EtOAc-soluble fraction (fraction A) was prepared from an extract of this ACR, it significantly decreased *iNOS* gene expression in primary cultured rat hepatocytes, in which the *iNOS* gene has been induced by IL-1β [12]. Four compounds were identified in ACR fraction A (Fig. 1). Among them, atractylodin markedly suppressed NO production and *iNOS* gene expression in IL-1β-treated hepatocytes (Table 2), whereas the sesquiterpenes hinesol, β-eudesmol, and (−)-α-bisabolol possessed much lower potency to suppress NO production. Previous reports demonstrated NO-suppressing potency of constituents of the root and rhizome of *Saposhnikovia divaricata* and *Glehnia littoralis* [34]. Falcarinol, another polyacetylene compound identified in this report, did not change NO production in hepatocytes [34]. Comparing chemical structures, a furan structure is present in atractylodin but not in falcarinol and may, therefore, be involved in the suppression of NO production and *iNOS* gene expression.

Atractylodin suppressed the expression of other genes that are involved in inflammation (Fig. 3). The plasma membrane may be permeable to atractylodin, which is a hydrophobic constituent. Atractylodin inhibited iNOS induction possibly by modulating various intracellular mechanisms via transcription factors, such as nuclear factor-κB (NF-κB) [8]. Phosphorylation of both NF-κB p65 subunit and an inhibitor of NF-κB α (IκB-α) may be involved in nuclear translocation and transactivation of NF-κB [16, 35]. However, Western blot analysis showed that atractylodin did not affect the phosphorylation of the serine residues of NF-κB p65 subunit or IκB-α (data not shown). In contrast, an electrophoretic mobility shift assay (EMSA) with nuclear extracts of hepatocytes showed that atractylodin decreased the DNA-binding activity of NF-κB (data not shown). Taken together, these results imply the possibility that atractylodin may suppress the NF-κB-mediated mechanism in hepatocytes.

Constituents in other crude drugs of Kampo medicine, for example, gomisin N in fruit of *Schisandra chinensis* [14], limonin in bark of *Phellodendron amurense* [15], and sakuranetin in bark of *Prunus jamasakura*, also suppress both *iNOS* and other proinflammatory genes [16]. All these constituents inhibited iNOS induction and affected the expression of proinflammatory genes at different levels, possibly by mechanisms via NF-κB [14–16]. Many IL-1β-inducible genes, including *iNOS*, *TNF*-*α*, *CCL2*, and *CCL20* genes, contain NF-κB-binding sites in their promoters, and NF-κB mediates the inducible expression of these genes [19, 28, 36]. Furthermore, signal transducer and activator of transcription 1α (STAT-1α) activated by Janus kinases (JAKs) may also be involved in the *iNOS* gene expression in most cells [8]. The involvement of the JAK/STAT-1α pathway in rat hepatocytes should be studied in the future.

LPS stimulates macrophages (e.g., RAW264.7 cells) to produce NO and proinflammatory cytokines, whereas rat hepatocytes do not respond to LPS [29]. Therefore, the effects of fraction A of the ACR fraction may be evaluated using LPS-treated macrophages. Indeed, *Atractylodes* extracts and α-bisabolol suppressed NO production in RAW264.7 cells, although the NO-suppressing potency was not shown [7, 10, 11]. According to previous studies using both RAW264.7 cells and rat hepatocytes [29], it is possible that the ACR fraction may show similar effects on iNOS expression in RAW264.7 cells.

Furthermore, the antiinflammatory fraction A of ACR extract improved the pathological findings in the kidney of HIGA mice, which is an IgA nephropathy model. Mesangial lesions in the glomeruli, i.e., mesangial proliferation associated with IgA deposition, become obvious in HIGA mice from age of 25 weeks [17]. Daily administration of ACR fraction A decreased these lesions (Fig. 5a). However, this administration did not significantly affect *iNOS* mRNA levels (Fig. 5c), and a nonspecific esterase stain revealed no marked infiltration of monocytes or macrophages in the kidney (data not shown). Because iNOS is primarily synthesized in macrophages and hepatocytes [8], these results seem to be plausible.

Serum creatinine and BUN concentrations are biomarkers that correlate with human renal function. However, no change in serum creatinine concentrations was observed in HIGA(−) mice (Table 3). Yoshimura et al. reported similar results for serum creatinine [31]. They also reported that BUN levels in HIGA mice were slightly higher than in the BALB/c(−) mice. In our study, BUN concentration in HIGA(−) mice was higher than in BALB/c(−) mice at age of 20 weeks, but lower at age of 30 weeks (Table 3). It is possible that urinary volume may reflect the excretion of BUN in the urine and affect its serum level.

The Kampo formula *Saireito*, which includes *Sojutsu*, shows antiinflammatory effects in rat hepatocytes [13]. When *Saireito* (TJ-114; kindly provided by Tsumura & Co., Tokyo, Japan) was administrated daily to HIGA mice, the pathological findings in the glomeruli of the kidney were not significantly improved (data not shown). It is possible that the dose of *Saireito* (1% powder in the diet) and the dose of *Sojutsu* constituents in *Saireito* were too low.

Although atractylodin in ACR fraction A may be involved in the improvement of HIGA mouse pathology, this remains to be confirmed. Recently, Fujitsuka et al. reported that administration of atractylodin increased ghrelin signaling and prolonged survival in *klotho*-deficient mice, which is a model of human aging [37]. A variety of pharmacological functions of atractylodin should be investigated. In addition, because atractylodin is present in the rhizomes of both *A. chinensis* and *A. lancea*, a future study on the *A. lancea* rhizome is also warranted. These studies will lead to effective use of *Sojutsu* for kidney disease.

## Conclusions

As an example of *Sojutsu*, the extract from the ACR was examined for its antiinflammatory properties. Using IL-1β-treated hepatocytes, atractylodin and three sesquiterpenes were identified as constituents in an EtOAc-soluble fraction of an ACR extract (fraction A). Because atractylodin effectively suppressed NO production and *iNOS* gene expression, it may be primarily responsible for the antiinflammatory activity of the ACR extract. Daily administration of the EtOAc-soluble fraction for 20 weeks improved the mesangial lesions in the renal glomeruli of IgA nephropathy model mice. Although further investigation is necessary to confirm the involvement of atractylodin in this improvement, this study provides a basis for developing the use of *Sojutsu* (*A. chinensis* and *A. lancea*) and atractylodin in the treatment of human kidney disease.

## Abbreviations

ITS1: Internal transcribed spacer 1
NO: Nitric oxide
iNOS: Inducible nitric oxide synthase
HIGA: High immunoglobulin A
IgA: Immunoglobulin A
EtOAc: Ethyl acetate
IL: Interleukin
NMR: Nuclear magnetic resonance
HPLC: High-performance liquid chromatography
TLC: Thin-layer chromatography
GC–MS: Gas chromatography–mass spectrometry
EI–MS: Electron ionization–mass spectrometry
PCR: Polymerase chain reaction
EF: Elongation factor 1α
LDH: Lactate dehydrogenase
ACR: *Atractylodes chinensis* rhizome
LCN2: Lipocalin 2
