# Correction to: Antiinflammatory constituents of *Atractylodes chinensis* rhizome improve glomerular lesions in immunoglobulin A nephropathy model mice

**DOI:** 10.1007/s11418-020-01405-w

**Published:** 2020-04-10

**Authors:** Toshinari Ishii, Tetsuya Okuyama, Nao Noguchi, Yuto Nishidono, Tadayoshi Okumura, Masaki Kaibori, Ken Tanaka, Susumu Terabayashi, Yukinobu Ikeya, Mikio Nishizawa

**Affiliations:** 1 Department of Biomedical Sciences, College of LifeSciences, Ritsumeikan University, Kusatsu, Shiga Japan; 2 College of Pharmaceutical Sciences, Ritsumeikan University, Kusatsu, Shiga Japan; 3 Research Organization of Science and Technology, Ritsumeikan University, Kusatsu, Shiga Japan; 4 Department of Surgery, Kansai Medical University, Hirakata, Osaka Japan; 5 Laboratory of Pharmacognosy and Medicinal Resources, Yokohama University of Pharmacy, Totsuka‑ku, Yokohama, Japan; 6 Center for Supporting Pharmaceutical Education, Daiichi University of Pharmacy, 22‑1 Tamagawa‑cho, Minami‑ku, Fukuoka, 815‑8511 Japan

## Correction to: Journal of Natural Medicines (2020) 74:51–64 10.1007/s11418-019-01342-3

The article Antiinflammatory constituents of *Atractylodes chinensis* rhizome improve glomerular lesions in immunoglobulin A nephropathy model mice, written by Toshinari Ishii, Tetsuya Okuyama, Nao Noguchi, Yuto Nishidono, Tadayoshi Okumura, Masaki Kaibori, Ken Tanaka, Susumu Terabayashi, Yukinobu Ikeya and Mikio Nishizawa was originally published Online First without Open Access. After publication in volume 74 issue 1, page 51–64 the author decided to opt for Open Choice and to make the article an Open Access publication. Therefore, the copyright of the article has been changed to © The Author(s) 2020 and the article is forthwith distributed under the terms of the Creative Commons Attribution 4.0 International License (http://creativecommons.org/licenses/by/4.0/), which permits use, duplication, adaptation, distribution and reproduction in any medium or format, as long as you give appropriate credit to the original author(s) and the source, provide a link to the Creative Commons license, and indicate if changes were made.

The original article was updated.

